# A “Campaign of Education”

**Published:** 1900-06

**Authors:** 


### TEXAS MEDICAL JOURNAL.

AUSTIN, TEXAS.

A MONTHLY JOURNAL OF MEDICINE AND SURGERY.

EDITED AND PUBLISHED BY

F. E. DANIEL, M. D., AND S. E. HUDSON, M. D.

Published Monthly at Austin, Texas, by Drs. Daniel and Hudson. Subscription price $1.00 a year in advance.

Eastern Representative: John Guy Monihan, St. Paul Building, 220 Broadway, New York City.

Official organ of the West Texas Medical Association, the Houston District Medical Association, the Austin District Medical Society, the Brazos Valley Medical Association, the Galveston County Medical Society, and several others.

### A "Campaign of Education."

THE TEXAS STATE MEDICAL ASSOCIATION, at its recent meeting at Waco, went into the subject of State Medicine and Public Hygiene pretty extensively, and all agreed that sanitary legislation, and medical legislation too, is a pressing necessity in Texas. The papers and addresses and the discussions on them are published herewith (except the paper of Dr. West on "The Maladministration of Medical Affairs in Texas," which appeared in our last issue). Those who are interested in the movement looking to securing co-operation by the people in the efforts of the medical profession to induce the Legislature to take up the subject are earnestly invited to read them. It will be seen that most of the speakers thought the best way to reach the legislators is through the people, before election of representatives; to bring to bear upon candidates such pressure as to get them pledged to sanitary reform, and a repeal of the obnoxious "practice act," which recognizes a diploma as a license to practice medicine. Our own views on this subject are set forth in the discussion. To the desired end it was determined to inaugurate a "campaign of education"; to make the voters understand that it is a duty the State owes every citizen—man, woman and child—to protect his health and life, as well against indiginous diseases—those which arise in our midst from ignorance or neglect of the simplest rules of sanitation, and which a knowledge of the causes would enable the authorities to prevent; as well from the ignorant "doctor,"—such, for instance, as one "decorated" by the late New York Medical College with a "diploma,"—as from imported diseases, against which all the "sanitary" machinery of the State is directed. The method of this campaign of education determined upon by the State Medical Association was the appointment of a special committee who are instructed to "compile, edit and publish the papers on the subject and the discussion on same, and to mail them to the voters of Texas." The committee consists of Drs. R. H. Harrison, Columbus, Texas; F. E. Daniel, Austin, Texas, and J. B. Massie, Houston, Texas. The work is under way, and it is contemplated to issue this matter in pamphlet form, of which a good many thousand copies will be printed—the exact number not yet agreed upon, and mail them out to every county in the State;—put them where they will "do most good." The Association is in a prosperous condition as to increased membership,—increased interest in association work, and financially. The Treasurer reports some $1200 or $1500 on hand, and the Association out of debt. Newspapers will seldom publish any medical or sanitary articles, unless paid, and the JOURNAL suggests that in addition to the pamphlets the leading dailies be furnished with the gist of this matter and paid to publish it. Many people will read such matter in the newspaper who would not read a pamphlet. It has been suggested that in order to interest politicians in the subject of sanitary and medical legislation the doctors shall "stand in with them" in the furtherance of some of their own schemes,—they all have schemes—bills they want passed—or something which will enable them individually to climb up the political ladder a little; but as the profession has no personal interest in getting the State to do what is clearly the State's duty to its people to do,—and their action is entirely disinterested, and for humanity, the suggestion does not strike us favorably. *This* might be done, however, and we ask our readers to consider the suggestion: Let each county send an influential medical man to the State democratic convention; let some of these get on the platform committee, and get in a "plank," pledging the party to sanitary and medical reform. In this way Texas was enabled, after repeated failures of work with the legislatures direct, to get the epileptic asylum at Abilene; only that it was not a medical man who secured a "plank" to that end in the convention at Galveston in 1898, but a lawyer, one who has interested himself in such matters, and was strongly impressed with the plea the doctors made in behalf of the unfortunates,—Hon. T. W. Gregory, of Austin, our immediate representative on the Board of University Regents. This is not generally known,—but it should be. Here we have a precedent,—a hint,—which might be adopted with advantage. For our own part we have very little hope of "educating the people" by any methods; we must "educate" the politicians—if possible.

